# The aggrecanopathies; an evolving phenotypic spectrum of human genetic skeletal diseases

**DOI:** 10.1186/s13023-016-0459-2

**Published:** 2016-06-28

**Authors:** Beth G. Gibson, Michael D. Briggs

**Affiliations:** Institute of Genetic Medicine, Newcastle University, Newcastle-upon-Tyne, NE1 3BZ UK; International Centre for Life, Central Parkway, Newcastle upon Tyne, NE1 3BZ UK

**Keywords:** Aggrecan, Osteochondritis dissecans, Chondrodysplasia, Cartilage, Skeletal dysplasia

## Abstract

The large chondroitin sulphated proteoglycan aggrecan (*ACAN*) is the most abundant non-collagenous protein in cartilage and is essential for its structure and function. Mutations in *ACAN* result in a broad phenotypic spectrum of non-lethal skeletal dysplasias including spondyloepimetaphyseal dysplasia, spondyloepiphyseal dysplasia, familial osteochondritis dissecans and various undefined short stature syndromes associated with accelerated bone maturation. However, very little is currently known about the disease pathways that underlie these aggrecanopathies, although they are likely to be a combination of haploinsufficiency and dominant-negative (neomorphic) mechanisms. This review discusses the known human and animal aggrecanopathies in the context of clinical presentation and potential disease mechanisms.

## Background

Long bones grow by a process of endochondral ossification and disruptions to this intricate and highly coordinated process result in a diverse group of genetic skeletal diseases (GSDs). GSDs are a clinically and genetically heterogeneous group of diseases are difficult to diagnosis and there are currently no treatments. This burden in pain and disability leads to poor quality of life and high healthcare costs. Current research efforts are focused on defining disease mechanisms and identifying potential therapeutic targets. One emerging group of GSDs are those resulting from defects in aggrecan, which is the primary proteoglycan component of the cartilage growth plate.

## Review

Aggrecan-related bone disorders (ORPHA364817) including:

- spondyloepimetaphyseal dysplasia, aggrecan type (ORPHA171866)
- macrocephally with multiple epiphyseal dysplasia and distinctive facies (OMIN607131)
- spondyloepiphyseal dysplasia, Kimberley type (ORPHA93283)
- familial osteochondritis dissecans (ORPHA251262)
- various idiopathic short stature phenotypes

## Rare skeletal diseases can provide new insight into fundamental disease mechanisms of cartilage degradation

Osteoarthritis (OA) is the most common form of arthritis. The World Health Organization estimates that 25 % of adults aged over 65 years suffer pain and/or disability from OA and it is ranked 12th for disease burden in the EU25 with 35–40 million people in Europe suffering from OA. OA is estimated to be 30–70 % genetic with strong environmental risk factors of ageing, obesity and joint trauma [1–3]. At the other end of the spectrum skeletal dysplasias are an extremely diverse and complex group of rare diseases that affect the development and homeostasis of the skeleton [4, 5]. There are more than 450 unique and well-characterised phenotypes and although individually rare, as a group of related orphan diseases, they have an overall prevalence of at least 1 per 4000 children. However, these genetically tractable skeletal dysplasias are a powerful tool for providing new insight into fundamental disease mechanisms of generalised cartilage degradation [6]. In this context, those skeletal dysplasias that result from defects in cartilage structural proteins, such as the collagens, proteoglycans and glycoproteins are of particular relevance for identifying mechanisms of accelerated cartilage degradation that will provide new insight into more common forms of OA [5, 6].

## Introduction to aggrecan; structure and function

**Fig. 1:**
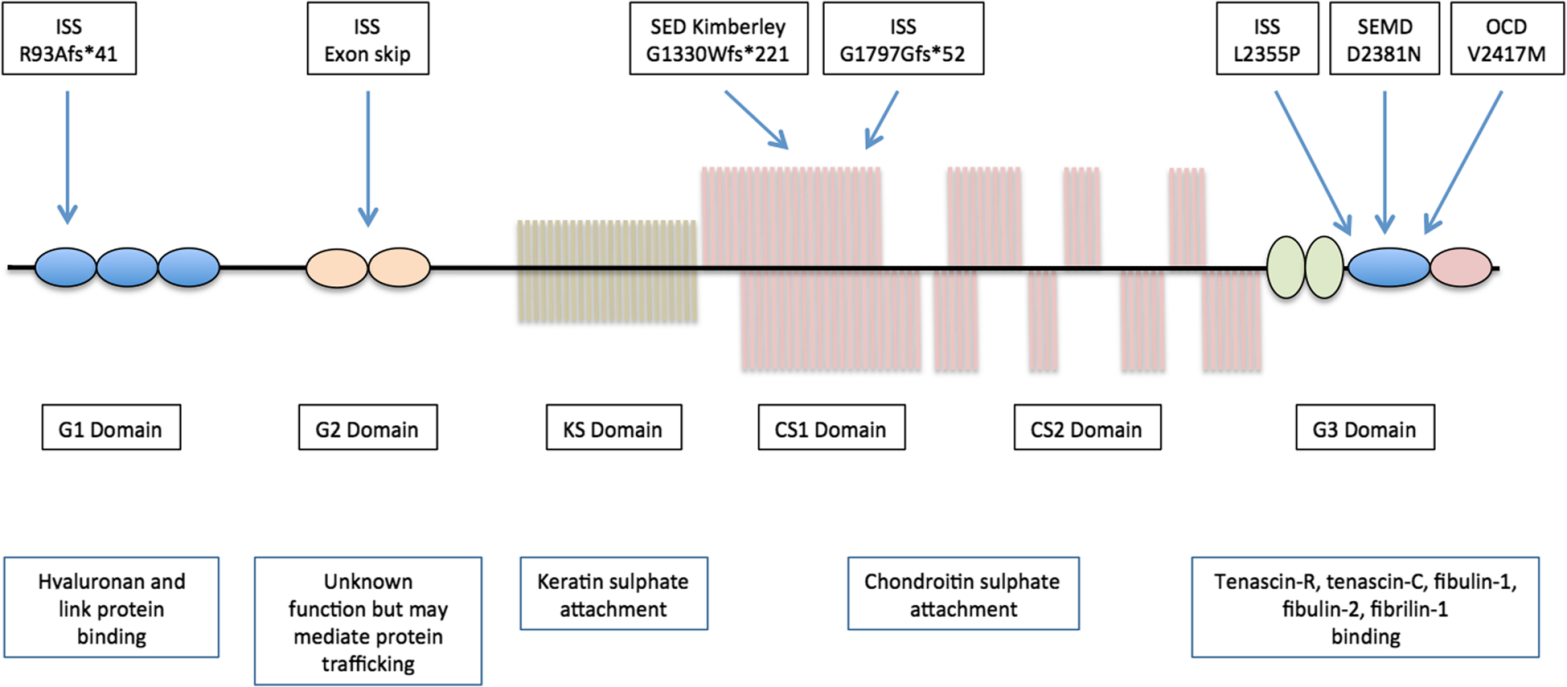
Schematic of the aggrecan showing the location of mutations and functions of the individual domains. Each of the seven human mutations is indicated (*top*) in the relevant domains (*middle*) that each has a specific function (*bottom*). Key: ISS = idiopathic short stature; SED = spondyloepiphyseal dysplasia; SEMD = spondyloepimetaphyseal dysplasia; OCD = osteochondritis dysplasia; G1 = globular domain 1; G2 = globular domain 2; G3 = globular domain 3; KS = keratin sulphate attachment domain; CS = chondroitin sulphate attachment domain

Aggrecan is a large chondroitin sulphated proteoglycan and the founding member of the lectican protein family, which also includes versican, brevican and neurocan [7]. It consists of a 250 kDa protein core with approximately 100 chondroitin sulphate glycosaminoglycan and 30 keratan sulphate chains attached to a large domain located between three globular domains. Aggrecan comprises an N-terminal domain, two globular domains (G1 and G2), two inter-globular domains, a selectin-like domain (G3) and a C-terminal domain (Fig. 1) [7, 8]. The two inter-globular domains act as chondroitin (CS) and keratin (KS) sulphate attachment regions [8]. The large G3 domain contains two type II (epidermal growth factor-like) repeats, a C-type lectin domain and a complement regulatory protein domain. Aggrecan is expressed in several tissues including those in the brain, but is a major structural component of cartilage. The fixed negative charge of this large proteoglycan is fundamental to cartilage because it attracts ions and water molecules, allowing the cartilage to withstand the high mechanical load found in the skeletal joint [7].

## The disease relevance of aggrecan mutations

The fundamental importance of aggrecan for cartilage development and homeostasis is well proven and was originally illustrated by several naturally occurring mutations such as the embryonically lethal chicken (*nanomelia*) [9] and mouse (*cmd*) [10]. Furthermore, Dexter bulldog dwarfism in cattle, which is a recessive form of dwarfism documented since the 19th Century, has two causative mutations in aggrecan [11]. More recently, genetic analysis of this most abundant of cartilage components is a fast emerging area of human connective tissue research, which will have a major influence on our understanding of both rare and common cartilage diseases. In particular, an allelic series of *ACAN* mutations have been identified that result in a broad phenotypic spectrum including spondyloepimetaphyseal dysplasia (SEMD) [12], spondyloepiphyseal dysplasia Kimberley type (SED) [13], familial osteochondritis dissecans [14] and various undefined short stature syndromes associated with accelerated bone maturation [15] (Table 1).

**Table 1:** Genetic and molecular findings in human aggrecanopathies and naturally occurring animal models

Gene mutation	Exon	Protein change	Domain	Molecular mechanism	Phenotype	Reference
c.272delA	3	Arg93Alafs*41	G1	Presumed haploinsufficiency	Idiopathic short stature	[15]
c.2026 + 1G > A	10	Not determined (but presumed exon skip)	G2	1.Presumed truncated protein	Idiopathic short stature	[15]
				2. Possible disruption to trafficking		
c.3986dupC	12	Gly1330Trpfs*221	CS1	Presumed haploinsufficiency	SED Kimberley type	[13]
c.5391delG	12	Gly1797Glyfs*52	CS2	Presumed haploinsufficiency	Dominant idiopathic short stature	[22]
c.7064 T > C	14	Leu2355Pro	G3	Presumed neomorphic	Short stature, accelerated bone maturation, and early growth cessation	[15]
c.6799G > A	15	Asp2267Asn	G3	Presumed neomorphic	Recessive SEMD, aggrecan type	[12]
c.7249G > A	16	Val2303Met	G3	Presumed neomorphic	Dominant osteochondritis dissecans, short stature, and early-onset osteoarthritis	[14, 17]
BD1 allele: 2266_2267insGGCA	11	Frame shift and introduction of PTC in exon 11 (amino acid position 914)		1. Null due to NMD of mRNA from mutant allele	Bulldog dwarfism (Dexter Cattle)	[11]
					Homozygous animals are embryonically lethal; heterozygous animals have short-limbed dwarfism with variable penetrance	
				2. Functional null due to production of truncated protein		
BD2 allele: −198C > T	1	Introduction of a novel start codon and translation of a 91 amino acid peptide with no resemblance to aggrecan; introduction of a PTC	n/a			
7 bp deletion leading to a PTC in exon 6.	5	Reduced mRNA levels in *cmd/cmd* (41 %) and *cmd/wt* (81 %) mice; truncated aggrecan molecule	G1	Functional and/or transcriptional null allele	*Cmd* (mouse)	[24–29]
					Homozygous mice are perinatally lethal; heterozygous mice show age-related spinal degeneration	
Large deletion	2–18	Unknown	G1-G3	Presumed null allele	*Cmd*-bc (mouse)	[30]
					Homozygous mice are perinatally lethal	
Glu1513Ter	10	Truncated aggrecan precursor that is retained in the ER; reduced mRNA levels	CS2	Presumed null allele	*Nanomelia* (chicken)	[9, 27, 29, 31, 33]
					Homozygous chicks are embryonically lethal with shortened and malformed limbs	
Unknown	n/a	Unknown	Unknown	Unknown	*CCI* (rat)	[35]
					Homozygous rats have short-limbed dwarfism, delayed anterior fontanel closing and insufficient cartilage calcification	

Details of the five aggrecan mutations that have been identified in families with a diverse range of human skeletal dysplasia and the naturally occurring animal models that have previously been studied

Key: *NMD* nonsense mediated degradation, *PTC* premature termination codon, *G1* globular domain, *G2* globular domain, *CS* chondroitin sulphate attachment domain, *SED* spondyloepiphyseal dysplasia, *SEMD* spondyloepimetaphyseal dysplasia, *BD* bull dog dwarfism, *cmd* cartilage matrix deficiency allele/mouse, *wt* wild type allele

*Indicates at which position the new reading frame encounters a translation termination (stop) codon stop

## Clinical and radiographic description of the human aggrecanopathies

### Spondyloepiphyseal dysplasia, Kimberley type (ORPHA93283; OMIM 608361)

A mild autosomal dominant condition in a single large family characterised by proportionate short stature (<5th percentile; males 141–162 cm and females 138–149 cm) with a stocky appearance and severe progressive osteoarthritis of the large weight bearing joints requiring joint replacement in middle age [16]. Radiographic features were minor and included prominent end plate irregularity and sclerosis of the vertebral bodies with mild and variable epiphyseal changes associated with delayed bone age. SED Kimberley results from a single-base pair insertion within the variable repeat region of exon 12 (c.3986dupC) that causes a frame shift of 212 amino acids and introduces a premature stop codon (p.Gly1330Trpfs*221) [13].

### Recessive spondyloepimetaphyseal dysplasia, aggrecan type (ORPHA93283: OMIM 612813)

An autosomal recessive condition in a single family characterised by extreme short stature (66–71 cm final height) with short necks, barrel chests and mild lumbar lordosis [12]. Craniofacial abnormalities include macrocephaly, severe mid-face hypoplasia and slightly low set ears. Radiographic examination showed irregular epiphyses and widened metaphyses, particularly at the knees. Spinal abnormalities included platyspondyly with multiple cervical-vertebral clefts, whilst in the hands there was proportionate brachydactyly with accessory carpal ossification centres. The carrier parents and half-sister in this family had adult heights of approximately 150 cm, whereas a non-carrier sister was 178 cm tall, suggesting that heterozygous carriers may have a mild proportionate short statue phenotype, similar to the familial osteochondritis dissecans described by Stattin and colleagues [17] or mild multiple epiphyseal dysplasia. SEMD, aggrecan type results from homozygosity for p.Asp2267Asn in the G3 domain of aggrecan [12].

### Macrocephaly with multiple epiphyseal dysplasia and distinctive facies (OMIN607131; OMIM 607131)

An autosomal recessive disease identified in a large multi-consanguineous family of Omani origin and clinically characterised by normal height but with *genu valgum* and dysmorphic features [18]. All four affected children had a head circumference >90^th^ centile and similar dysmorphic features including macrocephaly, frontal bossing, hypertelorism, a flat malar region and low set ears. Radiographs showed generalised epiphyseal dysplasia, which was more severe in the lower limbs. A second consanguineous family of Turkish origin with clinical findings of macrocephaly and facial abnormalities, and radiographic features of multiple epiphyseal dysplasia was reported by Karaer and colleagues [19]. They suggested that this was a second example of what they termed the “Al Gazali-Bakalinova Syndrome”.

Genetic mapping of the Omani MMEDF family identified a homozygous linked region at 15q26.1, but excluded aggrecan as a candidate gene due to heterozygosity for a polymorphism in exon 6 of *ACAN* [20]. However, these mapping data do not exclude the possibility of a recombination within *ACAN*, which might not be unexpected considering the large size of the gene (~75 kb). The genetic basis of this disease remains unresolved, but it could potentially be another member of the aggrecanopathies.

### Autosomal dominant osteochondritis dissecans, short stature, and early-onset osteoarthritis (ORPHA251262; OMIM 165800)

Osteochondritis dissecans (OCD) is characterised by the separation of an articular cartilage and subchondral bone fragment from the articular surface. The fragmented tissue may then become avascular and exist as a ‘loose body’ within the joint space [21]. OCD is the most common cause of loose bodies in adolescent individuals and has an approximate incidence of 1 in 2000 individuals. The aetiology of generalised OCD is unknown, but is likely to be a multifactorial combination of genetic variants, ischaemia and/or repetitive trauma.

The 15 year old proband in a large autosomal dominant family reported by Stattin and colleagues presented initially at the age of 6 with pain in the knees after exercise and a waddling gait [17]. Preliminary clinical examination confirmed an unrecognised skeletal dysplasia characterised by disproportionate short stature, low intervertebral discs in the thoracic and lumbar spine and OCD in both knees and a hip joint. A follow up analysis of the extended family confirmed that affected members had OCD in at least one joint and height ≤ −2 standard deviations when compared to the control population [mean final height for affected females was 148 cm (range 145–156) and for males was 156.5 cm (range 152–167)][17].

### Idiopathic short stature

Several cases of idiopathic short stature with causative mutations in aggrecan have recently been reported in the United States [15, 22]. Four families presented with autosomal dominant short stature, premature growth cessation and accelerated bone age maturation. Affected members of one family were heterozygous for a missense mutation (c.7064 T > C; p.Leu2355Pro) in exon 14 encoding the C-type lectin of the G3 domain [15]. This residue is highly conserved and the amino acid substitution is predicted to disrupt the function of aggrecan, but this has not been characterised in any detail. A second family were found to have a frameshift mutation (c.5391delG) in exon 12 (encoding the second inter-globular domain), which is predicted to introduce a premature stop codon (p.Gly1797Glyfs*52) [22]. Similarly, a third family were found to have a frameshift mutation (c.272delA) in exon 3 (encoding the G1 domain) that is predicted to produce a truncated protein (p.Arg93Alafs*41) [15]. Finally, a fourth family were found to have a novel base-pair substitution (c.2026 + 1G > A) in the highly conserved splice donor site of exon 10, which is predicted to result in incorrect splicing and the skipping of exon 10 [17]. The effect of this mutation on mRNA splicing was not investigated further, but might result in an in-frame deletion of 97 amino acids from the G2 domain of aggrecan.

## Aetiology and disease mechanisms in human aggrecanopathies

The 2015 revision of the “Nosology and Classification of Genetic Skeletal Disorders” provides a comprehensive catalogue of over 400 genetic disorders of the skeleton in 42 individual groups. The approach taken for the grouping of disorders was a combination of 1) a single gene or group of related genes 2) specific phenotypic feature, or 3) by radiological description. The aggrecanopathies are placed in a single group (number 6) based on genetic criteria alone. Recently, we have reviewed a select range of genetic skeletal diseases and have suggested alternative groupings based on common disease mechanisms. We now propose that there are potentially two different disease mechanisms in the aggrecan disease spectrum that are focused on either quantitative or qualitative defects [4, 5].

### Premature termination codons potentially resulting in truncated proteins or haploinsufficiency through nonsense mediated mRNA degradation

The heterozygous mutations identified in SED Kimberley (p.Gly1330Trpfs*221) [13] and some idiopathic short stature phenotypes (p.Gly1797Glyfs*52 and p.Arg93Alafs*41) [15] are predicted to cause a frame shift that introduces a premature termination codon. Premature termination codons usually result in nonsense-mediated degradation (NMD) of mRNA from the mutant allele [23] and therefore presumed haploinsufficiency for aggrecan. Unfortunately, cartilage mRNA was not available from any of these patients and it has not been possible to determine whether these human mutations cause NMD or allow translation of a truncated aggrecan protein that might be secreted into the cartilage extracellular matrix or retained and/or degraded. Retention and/or secretion into the ECM of a truncated aggrecan protein could possibly exert a dominant negative effect on cartilage structure and/or function.

### Dominant-negative (neo-morphic) missense mutations that disrupt cartilage structure and function

Interestingly, all three *ACAN* missense mutations, Leu2355Pro [15], Asp2267Asn and [12] Val2303Met [14] are located within the G3-domain (C-type lectin) and yet result in phenotypes that define the extensive clinical spectrum of the aggrecanopathies. For example, Asp2267Asn results in severe recessive SEMD characterised by extreme short stature (final adult height of only 66–71 cm) [12]; in contrast, patients with Val2303Met and Leu2355Pro present with milder phenotypes characterised by dominantly inherited osteochondritis dissecans (with mild short stature) [14] or short stature with accelerated bone maturation respectively [15].

It would therefore seem logical that this dichotomy in clinical severity is due to profoundly different disease mechanisms, and yet, biochemical analysis has revealed similar but modest pathological variances in vitro. For example, previous studies used patient cartilage and surrogate cell models to study the functional consequences of Asp2267Asn and Val2303Met [12, 14]. Both mutations allowed the secretion of mutant recombinant aggrecan G3-domains, but disrupted binding to several interacting partners such as fibulin-1 & -2 (Val2303Met) [14] and tenascin-R (Val2303Met) [14] or tenascin-C (Asp2267Asn) [12]. Neither of these biochemical studies therefore provided a mechanistic link between the specific aggrecan missense mutations and the resulting disease phenotypes of either severe short stature or cartilage instability. However, the apparent secretion of mutant aggrecan protein in vitro suggests that changes to the cartilage ECM are likely to be the underlying cause of disease pathology. These mutations will therefore provide a unique opportunity to study the role of abnormal cartilage ECM in disease initiation and progression, and how this pathology might relate to changes in cell phenotype. Unfortunately, the only well-characterised in vivo models previously studied are either the mouse *cmd* (cartilage matrix deficiency) [24–29] or the chick *nanomelia*, both of which are lethal and therefore of limited value for studying disease mechanisms in human aggrecanopathies. In the long term an allelic series of aggrecan transgenic mouse models will provide a valuable and unique resource to determine in vivo how spatially-related mutations can cause either profound disruptions to the cartilage growth plate and severely reduced bone growth (i.e. SEMD) or generalised cartilage instability that leads to osteochondritis dissecans.

## Existing animal models for studying disease mechanisms in aggrecanopathies

The only in vivo models of aggrecan pathologies previously studied are either the mouse *cmd*/*cmd*-bc (cartilage matrix deficiency) or the chick *nanomelia*, both of which are lethal as homozygotes and therefore of limited value as disease models for studying human aggrecanopathies (Table 1). In contrast, the heterozygote forms of these disorders are non-lethal and are therefore potentially useful as models; indeed, the biochemical analysis of tissues from all of these models has provided valuable insight into the role of aggrecan in cartilage development and homeostasis (Table 1).

### Cartilage matrix deficiency mouse (cmd)

The *cmd* mouse is the best studied of the aggrecan animal models [24–29] and results from a 7 bp deletion in exon 5 [27] that arose on an agouti background and is homozygous perinatal lethal. Mutant pups have abnormal tracheal cartilage and so respiratory failure occurs soon after birth. Other phenotypic features include disproportionate dwarfism, enlarged abdomen, short snout and tail, cleft palate and a protruding tongue. Studies have shown that the growth plate of these animals is disorganised and irregular; for example, there is a reduction in hypertrophic chondrocytes and the zones are no longer clearly defined with abnormal numbers of collagen fibrils [25, 29]. Aggrecan mRNA levels are reduced to 81 % and the expression patterns of other extracellular matrix molecules such as link protein, syndecan-3 and collagen II are all altered [28]. Interestingly, heterozygote mice are initially phenotypically normal, but develop proportional dwarfism by 28 days with age-associated spinal disc degeneration. The growth plates are irregular and there is a reduction in aggrecan mRNA levels to 41 % [20]. Cervical lordosis and thoraco-lumbar kyphosis develop and eventually lead to spinal misalignment. A primary lesion occurs in the intervertebral discs and there is decreased movement, which is often due to spastic paralysis in the hind limbs [20]. The mutant mice usually die within 19 months from starvation.

The related *Cmd-bc* mutation [30] spontaneously arose on a BALB/c GaBc background and, similar to the *Cmd* mouse, is perinatally lethal in homozygotes (the heterozygote has not been studied to date). Mutant pups have short-limbed dwarfism, an enlarged abdomen, protruding tongue, cleft palate and shortened snout. Although this phenotype is also due to a deletion, it arose through a non-homologous recombination event and so is far more extensive than *Cmd* causing the loss of exons 2–18 [30].

### The nanomelia chick model

The *nanomelia* chick is an autosomal recessive and embryonically lethal mutation [9]. Affected chick embryos display short-limbed dwarfism with a range of skeletal abnormalities. These irregularities include a large, brachycephalic head and an abnormal mandible and maxilla (causing a parrot-like beak) [9]. Further investigation revealed a transition (c.G4553T) that introduces a stop codon, premature termination and a truncated aggrecan precursor [31, 32], which is retained in the endoplasmic reticulum and does not undergo processing to mature aggrecan [33].

### Dexter bulldog dwarfism (Dexter cattle)

Dexter bulldog dwarfism is one of the oldest recorded naturally occurring animal genetic disorders and is fatal, resulting in the spontaneous mid to late-term abortion of homozygotes [34]. Homozygote calves display severe short-limbed dwarfism with a short vertebral column, marked vertebral platyspondyly, large abdominal hernia and extreme rib shortening. The head is disproportionately large with several abnormalities including midfacial retraction, relative prognathism, retruded muzzle and cleft palate. Heterozygote animals exhibit milder disproportionate dwarfism with rhizomelia, mild vertebral body irregularity and posterior wedging of vertebrae [34]. There are two causative mutations; a 4 bp insertion in exon 11 that causes a frameshift and premature stop codon (*BD1*) and a transition in exon 1 that introduces a novel ATG start codon and a large frameshift (*BD2*) [11]. The putative mutant protein resulting from the *BD2* mutation therefore has no resemblance to normal aggrecan. An in-depth analysis of Dexter bulldog dwarfism (*BD1*) has confirmed nonsense-mediated mRNA degradation from the mutant allele [11].

### Cartilage Calcification Insufficient rats (CCI)

The *CCI* rat mutation arose spontaneously in a Jcl-derived Sprague–Dawley colony [35]. The genetic cause of this rodent phenotype is currently unknown but it is thought to be an aggrecan synthesis disorder, inherited in an autosomal recessive manner. Mutant rats have short-limbed dwarfism with a short vertebral column and tail. The skull length is reduced with a delay in anterior fontanel closing. The bone spicules appear thicker and randomly organised, whilst the growth plate is irregular with a poorly formed secondary ossification centre and delayed endochondral ossification. Aggrecan expression in both articular cartilage and the growth plate is abnormal [35].

## Conclusions and future directions

Although the first human aggrecan mutation was genetically mapped in 2002 [36] and then molecularly defined in 2005 [13], remarkably, only six additional mutations have been identified and published in the intervening 10 years (Table 1). This limited identification of new mutations is most likely due to the difficulties in sequencing the relatively large and repetitive (i.e. intragenic VNTR) aggrecan gene using traditional Sanger approaches and a lack of multi-generation families for linkage studies. However, the adoption of next generation sequencing in both research and clinical diagnostics is starting to identify a plethora of *ACAN* variants of known and unknown functional relevance. The challenge now is to understand the functional significance of these novel variants, in particular missense mutations, and to determine disease mechanisms for the subsequent identification of therapeutic targets.

The study of specific human aggrecan variants has previously relied on surrogate cell model systems and a limited number of cartilage samples [12, 14], whilst the various naturally occurring animal models are not necessarily relevant for the equivalent human disease. However, recent research and technological initiatives and advances will help to readdress this imbalance. For example, the European Commission FP7-Heath funded project SYBIL (systems biology for the functional validation of genetic determinants of skeletal diseases) is currently generating and deep-phenotyping two mouse models of the aggrecanopathies that will ultimately help to determine disease mechanisms, whilst the use of genome editing will allow additional cell and mouse models to be generated and phenotyped in-depth as new variants of unknown significance are identified.

Defining disease mechanisms and identifying new therapeutic targets in the aggrecanopthies is likely to present a challenge, but it is tempting to speculate that they may fall into two broad categories focused on either quantitative or qualitative mechanisms. For the latter group, through studying a broad range of different genetic skeletal phenotypes resulting from dominant-negative (neo/anti-morphic) mutations in cartilage structural proteins, ER stress has been identified as a common disease mechanism [6]. Moreover, targeting ER stress as a therapeutic route by pharmacological agents is an exciting proposition that is gaining ever-greater momentum.

## Abbreviations

ACAN, aggrecan; CCI, cartilage calcification insufficient rats; CMD, cartilage matrix deficient; CS, chondroitin sulphate; ECM, extracellular matrix; G1-G3, globular domains; KS, keratin sulphate; MMEDF, macrocephaly with multiple epiphyseal dysplasia and distinctive facies; NMD, nonsense-mediated degradation; OA, osteoarthritis; OCD, osteochondritis dissecans; SED, spondyloepiphyseal dysplasia; SEMD, spondyloepimetaphyseal dysplasia 

## References

[1] Loughlin J (2015). Genetic contribution to osteoarthritis development: current state of evidence. Curr Opin Rheumatol.

[2] Yucesoy B, Charles LE, Baker B, Burchfiel CM (2015). Occupational and genetic risk factors for osteoarthritis: a review. Work.

[3] Aury-Landas J, Marcelli C, Leclercq S, Boumediene K, Bauge C: Genetic Determinism of Primary Early-Onset Osteoarthritis. Trends Mol Med. 2016;22(1):38-52. 10.1016/j.molmed.2015.11.006 26691295

[4] Warman ML, Cormier-Daire V, Hall C, Krakow D, Lachman R, LeMerrer M, Mortier G, Mundlos S, Nishimura G, Rimoin DL (2011). Nosology and classification of genetic skeletal disorders: 2010 revision. Am J Med Genet A.

[5] Briggs MD, Bell PA, Wright MJ, Pirog KA (2015). New therapeutic targets in rare genetic skeletal diseases. Expert Opin Orphan Drugs.

[6] Briggs MD, Bell PA, Pirog KA (2015). The utility of mouse models to provide information regarding the pathomolecular mechanisms in human genetic skeletal diseases: The emerging role of endoplasmic reticulum stress (Review). Int J Mol Med.

[7] Hardingham TE, Fosang AJ, Dudhia J (1994). The structure, function and turnover of aggrecan, the large aggregating proteoglycan from cartilage. Eur J Clin Chem Clin Biochem.

[8] Aspberg A (2012). The different roles of aggrecan interaction domains. J Histochem Cytochem.

[9] Landauer W (1965). Nanomelia, a Lethal Nutation of the Fowl. J Hered.

[10] Rittenhouse E, Dunn LC, Cookingham J, Calo C, Spiegelman M, Dooher GB, Bennett D (1978). Cartilage matrix deficiency (cmd): a new autosomal recessive lethal mutation in the mouse. J Embryol Exp Morphol.

[11] Cavanagh JA, Tammen I, Windsor PA, Bateman JF, Savarirayan R, Nicholas FW, Raadsma HW (2007). Bulldog dwarfism in Dexter cattle is caused by mutations in ACAN. Mamm Genome.

[12] Tompson SW, Merriman B, Funari VA, Fresquet M, Lachman RS, Rimoin DL, Nelson SF, Briggs MD, Cohn DH, Krakow D (2009). A recessive skeletal dysplasia, SEMD aggrecan type, results from a missense mutation affecting the C-type lectin domain of aggrecan. Am J Hum Genet.

[13] Gleghorn L, Ramesar R, Beighton P, Wallis G (2005). A mutation in the variable repeat region of the aggrecan gene (AGC1) causes a form of spondyloepiphyseal dysplasia associated with severe, premature osteoarthritis. Am J Hum Genet.

[14] Stattin EL, Wiklund F, Lindblom K, Onnerfjord P, Jonsson BA, Tegner Y, Sasaki T, Struglics A, Lohmander S, Dahl N (2010). A missense mutation in the aggrecan C-type lectin domain disrupts extracellular matrix interactions and causes dominant familial osteochondritis dissecans. Am J Hum Genet.

[15] Nilsson O, Guo MH, Dunbar N, Popovic J, Flynn D, Jacobsen C, Lui JC, Hirschhorn JN, Baron J, Dauber A (2014). Short stature, accelerated bone maturation, and early growth cessation due to heterozygous aggrecan mutations. J Clin Endocrinol Metab.

[16] Anderson IJ, Tsipouras P, Scher C, Ramesar RS, Martell RW, Beighton P (1990). Spondyloepiphyseal dysplasia, mild autosomal dominant type is not due to primary defects of type II collagen. Am J Med Genet.

[17] Stattin EL, Tegner Y, Domellof M, Dahl N (2008). Familial osteochondritis dissecans associated with early osteoarthritis and disproportionate short stature. Osteoarthr Cartil.

[18] al-Gazali LI, Bakalinova D (1998). Autosomal recessive syndrome of macrocephaly, multiple epiphyseal dysplasia and distinctive facial appearance. Clin Dysmorphol.

[19] Karaer K, Rosti RO, Torun D, Sanal HT, Guran S (2012). Macrocephaly with multiple epiphyseal dysplasia: a second example of Al Gazali-Bakalinova syndrome?. Genet Couns.

[20] Bayoumi R, Saar K, Lee YA, Nurnberg G, Reis A, Nur EKM, Al-Gazali LI (2001). Localisation of a gene for an autosomal recessive syndrome of macrocephaly, multiple epiphyseal dysplasia, and distinctive facies to chromosome 15q26. J Med Genet.

[21] Edmonds EW, Polousky J (2013). A review of knowledge in osteochondritis dissecans: 123 years of minimal evolution from Konig to the ROCK study group. Clin Orthop Relat Res.

[22] Quintos JB, Guo MH, Dauber A (2015). Idiopathic short stature due to novel heterozygous mutation of the aggrecan gene. J Pediatr Endocrinol Metab.

[23] Schweingruber C, Rufener SC, Zund D, Yamashita A, Muhlemann O (2013). Nonsense-mediated mRNA decay - mechanisms of substrate mRNA recognition and degradation in mammalian cells. Biochim Biophys Acta.

[24] Kimata K, Barrach HJ, Brown KS, Pennypacker JP (1981). Absence of proteoglycan core protein in cartilage from the cmd/cmd (cartilage matrix deficiency) mouse. J Biol Chem.

[25] Kobayakawa M, Iwata H, Brown KS, Kimata K (1985). Abnormal collagen fibrillogenesis in epiphyseal cartilage of CMD (cartilage matrix deficiency) mouse. Coll Relat Res.

[26] Kochhar DM (1985). Cellular expression of a mutant gene (cmd/cmd) causing limb and other defects in mouse embryos. Prog Clin Biol Res.

[27] Watanabe H, Kimata K, Line S, Strong D, Gao LY, Kozak CA, Yamada Y (1994). Mouse cartilage matrix deficiency (cmd) caused by a 7 bp deletion in the aggrecan gene. Nat Genet.

[28] Watanabe H, Nakata K, Kimata K, Nakanishi I, Yamada Y (1997). Dwarfism and age-associated spinal degeneration of heterozygote cmd mice defective in aggrecan. Proc Natl Acad Sci U S A.

[29] Wai AW, Ng LJ, Watanabe H, Yamada Y, Tam PP, Cheah KS (1998). Disrupted expression of matrix genes in the growth plate of the mouse cartilage matrix deficiency (cmd) mutant. Dev Genet.

[30] Bell L, Juriloff M, Harris MJ (1986). A new mutation at the cmd locus in the mouse. J Hered.

[31] Primorac D, Stover ML, Clark SH, Rowe DW (1994). Molecular basis of nanomelia, a heritable chondrodystrophy of chicken. Matrix Biol.

[32] Li H, Schwartz NB, Vertel BM (1993). cDNA cloning of chick cartilage chondroitin sulfate (aggrecan) core protein and identification of a stop codon in the aggrecan gene associated with the chondrodystrophy, nanomelia. J Biol Chem.

[33] Vertel BM, Grier BL, Li H, Schwartz NB (1994). The chondrodystrophy, nanomelia: biosynthesis and processing of the defective aggrecan precursor. Biochem J.

[34] Harper PA, Latter MR, Nicholas FW, Cook RW, Gill PA (1998). Chondrodysplasia in Australian Dexter cattle. Aust Vet J.

[35] Tanaka M, Watanabe M, Yokomi I, Matsumoto N, Sudo K, Satoh H, Igarashi T, Seki A, Amano H, Ohura K (2015). Establishment of a novel dwarf rat strain: cartilage calcification insufficient (CCI) rats. Exp Anim.

[36] Eyre S, Roby P, Wolstencroft K, Spreckley K, Aspinwall R, Bayoumi R, Al-Gazali L, Ramesar R, Beighton P, Gleghorn L (2005). Identification of a locus for a form of spondyloepiphyseal dysplasia on chromosome 15q26.1: exclusion of aggrecan as a candidate gene. J Med Genet.

